# Technology‐assisted revision knee arthroplasty reduces radiographic outliers compared with standard revision knee surgery: A systematic review

**DOI:** 10.1002/ksa.12748

**Published:** 2025-07-24

**Authors:** Matteo Innocenti, Filippo Leggieri, Simon N. van Laarhoven, Tommy de Windt, Roberto Civinini, Gijs G. van Hellemondt

**Affiliations:** ^1^ Department of Clinical Orthopaedics University of Florence Florence Italy; ^2^ Department of Orthopaedic Surgery Sint Maartenskliniek Nijmegen the Netherlands; ^3^ Orthopedisch Centrum Oost Nederland Orthopedic Center Hengelo the Netherlands

**Keywords:** joint line restoration, precision accuracy, robotic‐assisted revision total knee arthroplasty, robotic‐assisted revision unicompartmental knee arthroplasty, technology

## Abstract

**Purpose:**

The aim of this systematic review was to evaluate the outcomes and complications associated with technology‐assisted revision total knee arthroplasty (revTKA).

**Methods:**

A systematic search of PubMed, EMBASE, Web of Science and the Cochrane Library was conducted from inception to 31 October 2024. The inclusion criteria were experimental or observational studies with ≥10 patients undergoing robotic revTKA, evaluating clinical and/or radiological outcomes and/or complication rates. The exclusion criteria were isolated patellar revision, in vitro studies, letters to the editor, book chapters, conference papers, and studies without accessible full text. Each study was given a quality rating using the methodological index for non‐randomised studies (MINORS). The included studies were divided into those reporting revision from TKA and those from unicompartmental knee arthroplasty (UKA), for both qualitative and quantitative synthesis. Random‐effects meta‐analyses were performed where appropriate. Mean differences with 95% confidence intervals (CIs) were calculated for radiographic parameters. Heterogeneity was assessed using the *I* ^2^ statistic.

**Results:**

Across 20 studies (795 cases), 10 assessed revTKA and 10 examined revUKA. Of the revTKA studies, four were comparative cohorts, while among the revUKA studies, seven were comparative cohorts. Technology‐assisted revTKA showed fewer outliers in hip–knee–ankle angle (13.3% [95% CI, 8.7%–19.0%] vs. 26.1% [95% CI, 16.3%–38.1%]), superior component positioning within ±3° for the lateral distal femoral angle (88.4% [95% CI, 83.2%–92.4%] vs. 79.7% [95% CI, 68.8%–87.5%]) and for the medial proximal tibial angle (91.2% [95% CI, 86.3%–94.6%] vs. 82.6% [95% CI, 72.0%–89.8%]), and better joint line restoration (79.5% vs. 58.3% within 4 mm). Procedures required an additional 15–24 min. Complication rates were comparable between groups. For UKA revisions, outcomes were generally similar between technology‐assisted and conventional techniques, with mixed results on alignment accuracy and clinical scores.

**Conclusion:**

Technology‐assisted revTKA achieves optimal alignment parameters and reduces the occurrence of outliers compared with conventional techniques. However, these radiographic improvements do not consistently translate into enhanced clinical outcomes or reduced complication rates.

**Level of Evidence:**

Level IV.

**Abbreviations**
CAS: computer‐assisted surgery
FDA: U.S. Food and Drug Administration
HKA: hip–knee–ankle angle
LDFA: lateral distal femoral angle
MINORS: methodological index for non‐randomised studies
MPTA: medial proximal tibial angle
PICO: Patient, Intervention, Comparison and Outcome
PRISMA: Preferred Reporting Items for Systematic Reviews and Meta‐analyses
RA: robotic‐assisted
revTKA: revision total knee arthroplasty
revUKA: revision unicompartmental knee arthroplasty
TKA: total knee arthroplasty
TKR: total knee replacement
UKA: unicompartmental knee arthroplasty

## INTRODUCTION

Revision total knee arthroplasty (revTKA) is a demanding surgical procedure that must account for bone loss, the absence of anatomical landmarks, and ligament insufficiency following the removal of the index prosthesis. Achieving optimal outcomes in revTKA requires meticulous attention to the appropriate selection of implants, constraints and augmentations, as well as accurate placement of prosthetic components and restoration of neutral limb alignment [6, 45]. Furthermore, restoring joint line height is critical for preserving normal knee kinematics and biomechanics [14], while soft tissue management is paramount to ensure joint stability and long‐term recovery [5, 21, 23, 59, 63]. Accuracy in revTKA is essential because technical errors can compromise outcomes [15, 26, 50, 51, 58].

Robotic‐assisted (RA) implant placement has become a common procedure in primary knee joint arthroplasty [57]; however, its application in revision procedures is still in the early stages [61], which are traditionally performed with manual instrumentation based on a set of established conventions [7, 35, 47, 60]. For revision procedures, key alignment parameters include the hip–knee–ankle angle (HKA), which measures overall limb alignment; the lateral distal femoral angle (LDFA), reflecting femoral component coronal positioning; and the medial proximal tibial angle (MPTA), indicating tibial component coronal positioning [5, 28]. Robotic systems offer an alternative approach that facilitates real‐time control of multiple data points, enhances the understanding of and ability to act on patient‐specific anatomical features, improves the precision of bone cuts and implant positioning, optimises overall alignment and stability, and ultimately may contribute to improved clinical outcomes and enhanced long‐term implant survival [19, 30, 36, 38, 41]. Technology‐assisted revision knee arthroplasty may differ from conventional techniques in terms of radiographic alignment parameters, outlier rates, clinical outcomes, and complications.

The aim of this systematic review was to conduct a comprehensive analysis of the literature to evaluate the clinical and radiographic outcomes, as well as the complications, associated with technology‐assisted revision knee arthroplasties. Both revTKA and revUKA were included in this analysis because they represent different but related challenges in revision knee arthroplasty, where technology assistance may offer significant benefits. RevTKA addresses extensive bone loss and complex deformities, while revUKA‐to‐TKA conversion presents a distinct challenge, requiring the use of conservative primary implants despite potentially missing crucial anatomical landmarks [4, 31, 44]. By analysing both procedures, the review aimed to provide a comprehensive overview of technology applications across the spectrum of revision knee arthroplasty.

## MATERIALS AND METHODS

This systematic review adheres to the Preferred Reporting Items for Systematic Reviews and Meta‐Analyses (PRISMA) guidelines [42]. Initial searches on PROSPERO revealed no ongoing or completed systematic reviews on the designated topic. The current review was not listed on PROSPERO. A comprehensive search of electronic databases, including PubMed, EMBASE, Web of Science and the Cochrane Library, was conducted to identify relevant studies. The inclusion criteria were limited to published articles from inception to 31 March 2025. The combination of search terms and the research question employed to systematically retrieve pertinent studies can be found in the Supplementary Materials. The following search terms and Boolean operators were used: (((‘total knee replacement’ OR ‘total knee arthroplasty’ OR ‘unicompartmental knee replacement’ OR ‘unicompartmental knee arthroplasty’ OR ‘partial knee replacement’ OR ‘partial knee arthroplasty’ OR TKA OR TKR OR UKA) AND revision) AND (robot OR ‘robotic assisted’ OR ‘robot assisted’ OR navigation OR navigator OR mako OR rosa OR cori OR mazor OR velys OR navio OR excelsiusGPS OR skywalker OR computer OR ‘computer assisted’ OR CAS OR RA OR accelerometer)).

### Eligibility criteria

The articles selected for inclusion in the report were further filtered based on the following eligibility criteria, as outlined in the PICO question (provided in Supporting Information S1). For inclusion, a study had to meet the following criteria: a randomised controlled trial or observational analysis involving more than 10 patients undergoing revision knee arthroplasty, evaluating clinical and/or radiological outcomes or complication rates. The exclusion criteria were in vitro studies, letters to the editor, previous systematic reviews, book chapters, conference papers, and studies for which the full text was not accessible. Revision arthroplasty was defined as any operation in which the femoral and/or tibial component was exchanged. Polyethylene exchange or secondary patellar resurfacing without component exchange was not considered relevant for this review.

### Study selection

Initially, Zotero (Roy Rosenzweig Center for History and New Media, 2016) was employed to remove duplicate publications. Two independent reviewers conducted a thorough screening of references based on titles and abstracts. Full texts deemed eligible for inclusion were then acquired. Following the initial screening, the selected articles were further examined for relevant literature within their citation sections. In cases where the independent reviewers disagreed on whether to include or exclude a particular reference, a final decision was made by a senior author to resolve any discrepancies.

### Data extraction

From each included study, two independent reviewers extracted data, including the study design, sample size, follow‐up duration, surgery time, type of technology used, clinical and radiological outcomes, failure and complication rates, and a concise narrative description of the results. The included studies were divided into those reporting revision from TKA and those from UKA.

### Risk‐of‐bias assessment

Each study was given a quality rating using the Methodological Index for Non‐Randomised Studies (MINORS) to assess cohort studies and case series, respectively [53].

### Statistical analysis

For comparative studies, random‐effects meta‐analyses were performed where appropriate. Mean differences with 95% confidence intervals (CIs) were calculated for alignment parameters. Heterogeneity was assessed using the *I* ^2^ statistic, quantifying the percentage of total variation across studies due to heterogeneity rather than chance. *I* ^2^ values were interpreted according to the Cochrane Handbook guidelines: 0%–40% as potentially unimportant heterogeneity, 30%–60% as moderate heterogeneity, 50%–90% as substantial heterogeneity and 75%–100% as considerable heterogeneity. For all pooled analyses, the 95% CIs for the *I* ^2^ values were calculated to provide insight into the precision of the heterogeneity estimate [13]. For revTKA studies, pooled analyses included alignment measurements (HKA, LDFA and MPTA) and outlier frequencies. Because of significant heterogeneity in study designs and outcome measures among revUKA studies, a narrative synthesis approach was adopted. All analyses were performed using R Statistical Software v4.1.2.

## RESULTS

**Figure 1:**
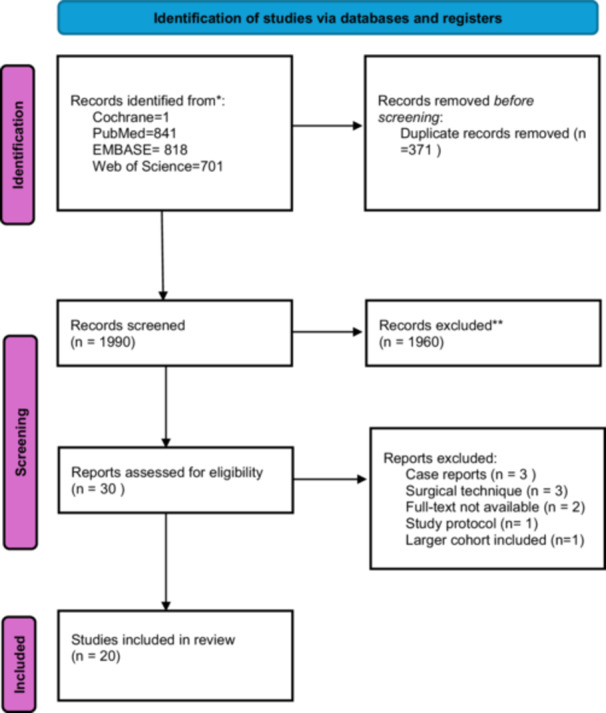
The PRISMA flowchart displays the search and selection process of the included studies. PRISMA, Preferred Reporting Items for Systematic Reviews and Meta‐Analyses.

The initial search across the databases generated 2361 studies. The PRISMA 2020 flowchart used to summarise the research strategy is shown in Figure 1 [42]. Ten articles were excluded: three articles describing case reports [20, 27, 54], three articles describing surgical techniques [11, 16, 32], two unavailable full‐texts [2, 17], one study protocol [34] and one partial sample of an already included population [48].

Finally, a total of 20 studies were included, of which 10 evaluated technology‐assisted revTKA [1, 9, 18, 27, 33, 37, 39, 43, 52, 55] and 10 focused on technology‐assisted revUKA [3, 8, 10, 22, 24, 29, 31, 49, 56, 62]. Among the revTKA articles, four included cohort studies [1, 18, 33, 43] (Table 1) and six presented one‐cohort‐only case series [9, 27, 37, 39, 52, 55] (Table 2). Among the revUKA articles, seven included cohort studies [10, 22, 24, 31, 49, 56, 62] (Table 3) and three presented one‐cohort‐only case series [3, 8, 29] (Table 4).

**Table 1:** Cohort studies evaluating revision TKA.

Author, year	Design	Cohorts	Size of population (knees)	Age	Follow‐up (months)	Cause for revision	Technology	Surgery time (min)	Clinical outcomes	SMD
Alling, 2024	Prospective matched cohorts	Navigated revTKA B = Standard revTKA 23	*A* = 62 *B* = A	*A* = 63.8 ± 9.3 *B* = 60.3 ± 13.7	N/A	Loosening: 31 (50%) Malalignment: 24 (38.7%) Instability: 14 (22.6%)	Navitrack	*A* = 282 ± 46 *B* = 258 ± 46	N/A	N/A
Massin, 2008	Unmatched cohorts	A= Navigated B = Standard	*A* = 19 *B* = 10	66 (range: 20–80)	11	A = Unicompartmental: 5 (26.3%) Aseptic loosening: 3 (15.8%) Instability: 2 (10.5%) Wear: 6 (31.6%) PJI: 3 (15.8%) Combination: 1 (5.3%) B = Unicompartmental: 2 (20%) Aseptic loosening: 4 (40%) Instability: 1 (10%) Wear: 1 (10%) PJI: 1 (10%) Combination 1 (10%)	PRAXIM Medivision	*A* = 200 min (range, 120–300 min) *B* = 180 min (range, 145–210 min)	IKS	KSS Knee Scores: A: Satisfactory: 16 (84.2%) Fair: 1 (5.3%) Poor: 2 (10.5) B: Satisfactory: 9 (90%) Poor: 1 (10%)
Jenny, 2010	Prospective unmatched cohorts	A = Navigated B = Standard	*A* = 50 *B* = 36	70.6 ± 7.7	N/A	Aseptic loosening: 52 (60.5%) Infection: 32 (37.2%)	Orthopilot	N/A	N/A	N/A
Perlick, 2005	Prospective unmatched cohorts	A = Navigated B = Standard	*A* = 50 *B* = 50	73.8 (range: 62–84)	N/A	Loosening *A*: 15 (52%) *B*: 16 (68%) Instability *A*: 6 (32%) *B*: 7 (32%) Wear *A*: 4 (16%) *B*: 2 (12%)	Vector vision	*A* = 104 min (±19 min) *B* = 89 min (±16 min)	N/A	N/A

Abbreviations: KSS, Knee Society Score; PJI, periprosthetic joint infection; revTKA, revision total knee arthroplasty; SMD, standardised mean difference; TKA, total knee arthroplasty.

**Table 2:** Case series evaluating revision total knee arthroplasty (revTKA).

Author, year	Size of population (knees)	Age	Follow‐up (months)	Cause for revision	Technology	Surgery time (min)	Clinical outcomes	SMD	Complications
MacAskill, 2024	25	67.08 ± 7.86	N/A	Instability: 13 cases (52.0%) Loosening: 7 cases (28.0%) PJI: 5 cases (20.0%)	MAKO	N/A	N/A	N/A	None
Cochrane, 2023	115	65 (range, 43–88)	13 (range, 3–51)	Loosening: 42 (37%) Instability: 37 (32%) Failed UKA: 15 (13%) PJI: 9 (8%) Stiffness: 7 (6%) Extensor mechanism failure: 5 (4%)	CORI	186 (range, 58–466)	PROMIS scoring system	Pain: −1.08 PROMIS physical function: 0.06 PROMIS pain interference: −0.38 PROMIS depression: −0.30	Reoperations: 5 MUA: 2
Ochs, 2014	18	57.0 ± 8.0	21.9 ± 6	Instability: 11 (61.1%) Loosening: 6 (33.3%) Stiffness: 1 (5.6%)	Orthopilot	N/A	Very satisfied: 3 (16.67%) Satisfied: 9 (50%) Sufficient: 3 (16.67%) Unsatisfied: 3 (16.67%) KSS Knee: 58.4 ± 16.7 KSS Functional: 65.6 ± 24.0 KOOS Pain: 50.2 ± 27.4 Symptoms: 59.5 ± 22.1 ADL (Activities of Daily Living): 53.8 ± 25.8 Sport/Recreation: 14.4 ± 19.5 QOL (Quality of Life): 39.2 ± 20.9	N/A	Navigation‐associated: 0 (0%) Osteitis pin track: 1 (5.6%) Wound leakage: 2 (11.1%) Loosening: 1 (5.6%) Patellar Pain: 2 (11.1%) Overall: 6 (33.3%)
Sikorski, 2004	14	77.6 ± 7.4	N/A	Wear: 11 (68.8%) Malalignment: 3 (18.8%) Lateral compartment OA: 1 (6.2%) Loosening: 1 (6.2%)	Stryker navigation system	N/A	ROM > 90° (100%)	N/A	Residual pain 1 (7.14%)
Thielemann, 2007	46	72 years (range, 67‐81)	N/A	Infection: 9 (19.6%) Loosening: 17 (37.0%) Malalignment: 20 (43.5%)	OrthoPilot	N/A	Flexion > 90°: 46 (100%)	N/A	Residual pain: 5 (10.87%)
Ngim, 2023	23	69.7 (range, 57–84)	10.4 (range, 6–18)	revTKA: 12 (63.2%) Loosening: 11 (57.9%) Instability: 1 (5.3%) revUKA: 4 (21.1%) Wear: 2 (10.5%) Loosening: 2 (10.5%)	MAKO	N/A	ROM Extension 1.5 (range, 0–5) Flexion 114° (range, 100–130)	N/A	None

Abbreviations: KOOS, Knee injury and Osteoarthritis Outcome Score; KSS, Knee Society Score; MUA, manipulations under anaesthesia; OA, osteoarthritis; PJI, periprosthetic joint infection; revUKA, revision unicompartmental knee arthroplasty; ROM, range of motion; SMD, standardised mean difference; UKA, unicompartmental knee arthroplasty.

**Table 3:** Cohorts studies evaluating revision unicompartmental knee arthroplasty (revUKA).

Author	Design	Knees	Age (years)	Follow‐up (months)	Diagnosis	Implant	Surgery time	Cohorts	Clinical outcomes	Complications
Mancino, 2024	Prospective matched cohorts	*A* = 16 *B* = 35	*A* = 72 (range, 57–85) *B* = 69 (range, 56–80)	*A* = 21 (range, 6–36) *B* = 19 (range, 6–48)	OA progression: 10 (62%) Pain: 2 (13%) Instability: 2 (13%) Loosening: 1 (6%) Valgus collapse: 1 (6%)	MAKO	*A* = 127 ± 18 *B* = 108 ± 24	A = Robotic UKA to revTKA B = primary TKA	ROM A = Preop: 112 ± 15 Postop: 119 ± 9 B = Preop: 108 ± 20 Postop: 118 ± 10 SMD A = FJS: 6.15 OKS: 5.11 B= FJS: 5.89 OKS: 5.82	No difference between Robotic and Standard A = Intraoperative tibial fracture 1 No radiolucent lines in either group No implant subsidence in either group
Confalonieri, 2010	Matched cohorts	*A* = 22 *B* = 22	*A* = 71.8 (range, 62–83) *B* = 73.6 (range, 66–81)	*A* = 42.7 ± 16.7 (range, 13–66) *B* = 48.4 ± 13.9 (range, 14–65)		OrthoPilot	*A* = 142.3 ± 13.7 (range, 85–132) *B* = 98.8 ± 11.8 (range, 80–122)	A = navigated from UKA B = standard from UKA	KSS Knee *A* = 80.04 ± 5.2 (range: 74–88) *B* = 77.9 ± 4.5 (range: 73–87) KSS‐FS *A* = 82.3 ± 8.9 (range: 70–100) *B* = 77.8 ± 8.2 (range: 69–90)	None
Lachance, 2023	Retrospective unmatched cohorts	*A* = 11 *B* = 10 *C* = 10 *D* = 17	57 ± 8	At least 1	Loosening (60%) Instability (10%) OA progression (10%) Poly dislocation (4%) PJI (2%), Other (14%)	MAKO	N/A	A = Manual to manual (11 knees) B = Manual to robotic (10 knees) C = Robotic to manual (10 knees) D = Robotic to robotic (17 knees)	ROM Extension *A* = 1.25 ± 2.5 *B *= 0 ± 0 *C* = 2.5 ± 7.07 *D* = 0.91 ± 3.02 Flexion *A* = 118.75 ± 6.29 *B* = 125 ± 7.07 *C* = 116.88 ± 13.35 *D* = 124.09 ± 6.64	Complications were documented in 10% of patients, with the highest in the manual‐robot group (18%). One robotic‐manual conversion required two MUAs, while one patient undergoing manual‐robotic and one robotic‐robotic needed MUA. One manual‐manual patient required revision surgery due to infection.
Lee, 2018	Retrospective propensity score matched cohorts	*A* = 15 *B* = 45	*A* = 69.3 ± 8.9 *B* = 67.5 ± 14.3	*A* = 44.2 ± 2.4 *B* = 41.6 ± 8.4	Aseptic loosening: 6 (40%) OA progression: 3 (20%) Wear: 5 (33.3%) Bearing dislocation: 1 (6.7%)	OrthoPilot		A = revUKA B = primary TKA	HSS score WOMAC score KSS score Feller score	N/A
Tuecking, 2021	Matched cohort	*A* = 20 *B* = 20	*A* = 62.4 ± 10.2 *B* = 68.9 ± 9.25	N/A	N/A	NAVIO	*A* = 76.0 *B* = 69.6	A = revUKA B = primary TKA		None
Yun, 2020	Unmatched cohorts	*A* = 17 *B* = 17	*A* = 70 (range, 42–82) *B* = 72 (range, 54–88)	*A* = 6.1 (range, 2–12) *B* = 1 (range, 1–2)	N/A	MAKO	*A* = 85 min (range 73–95) *B* = 83 min (range 64–96)	N/A	None	None
Saragaglia, 2015	Matched cohort	*A* = 23 navigated *B* = 23	*A* = 75.4 ± 8.5 (range, 55–90) *B* = 74.3 ± 8.5 (range, 53–93)	49.3 ± 34.7 vs. 118 ± 58.5	A = Loosening: 11 (47.8%) OA progression: 6 (26.1%) Wear: 3 (13%) Pain: 3 (13%) B = Loosening: 13 (56.5%) OA progression: 3 (13%) Wear: 5 (21.7%) Instability: 1 (4.3%) Pain: 1 (4.3%)	Orthopilot	N/A	A = Navigated revUKA B = Standard revUKA	A = Knee flexion: 114.3 ± 10.6° (range, 100–140°) KSS: Knee: 86 ± 6 (range, 72–100) Function: 91.5 ± 16.6 (range 80–100) B = Knee flexion: 110 ± 11.5° (range, 90–120°) KSS Knee 85 ± 14 (range, 50–100) FS: 80 ± 16 (range, 50–100)	N/A

Abbreviations: FJS, Forgotten Joint Score; HSS, Hospital for Special Surgery; KSS, Knee Society Score; MUA, manipulations under anaesthesia; OA, osteoarthritis; OKS, Oxford Knee Score; PJI, periprosthetic joint infection; revTKA, revision total knee arthroplasty; ROM, range of motion; SMD, standardised mean difference; TKA, total knee arthroplasty; UKA, unicompartmental knee arthroplasty; WOMAC, Western Ontario and McMaster Universities Osteoarthritis Index.

**Table 4:** Case series evaluating revision unicompartmental knee arthroplasty (revUKA).

Author, year	Design	Knees	Age	Follow‐up	Diagnosis	Implant	Surgery time	Clinical outcomes	Complications
Magruder, 2024	Prospective single‐centre study	44	72 years (range, 43–91)	1.8 years (range, 1–6.6)	Osteoarthritis progression: 33 (75.0%) Aseptic loosening: 7 (16.0%) Unspecified pain: 2 (4.5%) Polyethylene wear: 1 (2.3%) Prosthetic joint infection (PJI): 1 (2.3%)	MAKO	N/A	KOOS JR score: Increased from 48.1 to 68.7 r‐WOMAC score: Decreased from 25.7 to 10.6	PJI: 2 (4.5%) Aseptic loosening of femoral component: 1 (2.3%) Superficial surgical site infection requiring irrigation and debridement: 1 (2.3%)
Chatain, 2012	Prospective single‐centre study	20	N/A	Range: 2–7	Wear: 4 (20%) Subsidence: 2 (10%) Instability: 1 (5%) Pain: 6 (30%) Loosening: 6 (30%) Infection: 1 (5%)	Amplivision	N/A	KSS Knee median: 89–100 KSS FS median: 90 (70–100) Satisfied/very satisfied: 19 (95%) Disappointed: 1 (5%) ROM mean: 115° (90–140°)	None
Andriollo, 2024	Prospective single‐centre study	35 knees	69.7 ± 7 years	31.3 ± 12.1 months	OA progression: 28 (80%) UKA failure: 7 (20%) Aseptic loosening: 4 (11.4%) Painful hypercorrection: 2 (5.7%) Patellar instability: 1 (2.9%)	ROSA	116.1 ± 19.6 min	OKS improved from 31.4 to 41.5 WOMAC improved from 53.5 to 17.8 FJS‐12 improved from 47.3 to 80.7 ROM improved from 105.3° to 119.4°	1 poly revision for stiffness

Abbreviations: FJS, Forgotten Joint Score; KOOS JR, Knee injury and Osteoarthritis Outcome Score for Joint Replacement; KSS, Knee Society Score; OKS, Oxford Knee Score; ROM, range of motion; UKA, unicompartmental knee arthroplasty; WOMAC, Western Ontario and McMaster Universities Osteoarthritis Index.

### revTKA

Across 10 studies, 397 knees were treated using technology‐assisted techniques [1, 9, 18, 27, 33, 37, 39, 43, 52, 55]. Among these, 181 technology‐assisted revTKAs were compared with 69 conventional revTKAs [1, 18, 33, 43].

Interestingly, the data reflect the historical evolution of the technology used in revTKA. Studies from 2004 to 2010 employed basic first‐generation navigation systems (Navitrack, PRAXIM) [33, 43, 52, 55]. Those from 2010 to 2020 used more advanced navigation technology (Vector Vision, Orthopilot) [18, 40], while studies from 2020 to the present used robotic assistance (MAKO, CORI) [9, 37].

Consistently across these studies, computer‐assisted procedures required more time than conventional procedures, with the additional time ranging from 15 to 24 min [1, 33, 43].

Pooled data from comparative studies of postoperative HKA, LDFA, MPTA and joint line measurements are shown in Table 5. No complications were reported in the cohort studies; however, it remains unclear whether this reflects an actual absence of complications or a limitation in reporting. Complications from the case series are presented in Table 2.

**Table 5:** Radiological outcomes in technology‐assisted versus conventional revision knee arthroplasty.

Outcome measure	Technology‐assisted	Conventional	Difference	Notes	References
Mean HKA angle	180.8 ± 2.5° (95% CI: 180.4–181.2°)	179.7 ± 3.1° (95% CI: 178.9–180.5°)	1.1° (95% CI: 0.3–1.9°)	Technology‐assisted showed better accuracy	[18, 31, 33, 34]
HKA outliers (>180 ± 3°)	13.3% (95% CI: 8.7%–19.0%)	26.1% (95% CI: 16.3%–38.1%)	12.8% fewer outliers	Technology‐assisted had fewer alignment outliers	
Mean LDFA	90.3 ± 2.8° (95% CI: 89.8–90.8°)	89.7 ± 3.4° (95% CI: 88.9–90.5°)	0.6° (95% CI: 0.2–1.0°)	Technology‐assisted showed slight improvement	[1, 18, 27, 33, 43, 52]
LDFA within ±3° of neutral	88.4% (95% CI: 83.2%–92.4%)	79.7% (95% CI: 68.8%–87.5%)	8.7% more within target	Technology‐assisted had better femoral component alignment	
Mean MPTA	90.1 ± 1.7° (95% CI: 89.7–90.5°)	89.5 ± 2.4° (95% CI: 88.9–90.1°)	0.6° (95% CI: 0.3–0.9°)	Technology‐assisted showed better tibial alignment	[18, 27, 33, 39, 52]
MPTA within ±3° of neutral	91.2% (95% CI: 86.3%–94.6%)	82.6% (95% CI: 72.0%–89.8%)	8.6% more within target	Technology‐assisted had better tibial component alignment	
Joint line restoration					
Excellent (<4 mm elevation)	79.50%	58.30%	21.2% improvement	Technology‐assisted showed better joint line restoration	[43]
Moderate (4‐8 mm elevation)	17.20%	33.40%	16.2% fewer cases	Technology‐assisted had fewer moderate deviations	
Poor (>8 mm elevation)	3.30%	8.30%	5.0% fewer cases	Technology‐assisted had fewer poor outcomes	

Abbreviations: CI, confidence interval; HKA, hip–knee–ankle; LDFA, lateral distal femoral angle; MPTA, medial proximal tibial angle.

Overall, no authors reported meaningful clinical superiority of technology‐assisted surgery compared with the conventional [1, 33, 43] except for Jenny et al. [18], who found optimal global component positioning with improved accuracy in the navigated group.

### revUKA

Several studies included in this review focused on comparing technology‐assisted revUKA to primary robotic TKA procedures [24, 31, 56], while others examined the outcomes of robotic or navigated revUKA compared to conventional revUKA [10, 48, 62]. One study grouped its cohort based on both the type of initial UKA procedure (manual vs. robotic) and the subsequent TKA technique (manual or robotic) in a ‘two by two’ comparison [22].

Across the 10 included studies, 175 knees were evaluated in the cohort studies and compared to a total of 124 knees in the control groups [10, 22, 24, 31, 49, 56, 62]. Additionally, the overall sample size across the case series accounted for 99 knees [3, 8, 29].

The heterogeneity of the data precluded quantitative synthesis of the results, necessitating a narrative approach instead. The MAKO Robotic System® (Stryker) was the most commonly used platform, being preferred in four studies [22, 29, 31, 62], followed by the OrthoPilot System® (Aesculap AG) in three [10, 24, 48] and the NAVIO System® (Smith & Nephew) [56], the Amplivision® (Amplitude Ortho) [8] and the ROSA® (Zimmer) [3] in one each.

Regarding surgery times reported in the included studies, more recent articles [56, 62] demonstrated smaller time differences between technology‐assisted and conventional techniques, whereas earlier studies [10] reported larger discrepancies. Overall, surgery times varied between studies, ranging from 76 to 142 min, with additional time required for computer‐assisted or RA procedures ranging from 2 to 44 min. The overall complication rates were similar between computer‐assisted and control groups, as shown in Table 3.

Most studies demonstrated no significant differences between navigation‐assisted or RA platforms and conventional techniques in clinical or radiographic outcomes. Mancino et al. [31], Lee et al. [24] and Tuecking et al. [56] reported comparable outcomes, with no differences between technology‐assisted revUKAs (conversion to TKA) and primary TKAs. Confalonieri et al. [10], Saragaglia et al. [48] and Yun et al. [62] reported mixed results. Confalonieri et al. [10] demonstrated the superiority of navigation in joint line restoration, alignment accuracy, clinical scores, and reduced blood transfusion requirements for navigated procedures, albeit with a longer operative time than in conventional revUKA.

### Risk of bias

The tables showing the results of the risk‐of‐bias assessment are provided in Supporting Information S1.

## DISCUSSION

This systematic review and meta‐analysis comprehensively examined the outcomes of technology‐assisted revision knee arthroplasty, analysing both revTKA and revUKA procedures. To date, this is the first systematic review to evaluate the outcomes of revision knee surgery performed using technology‐assisted platforms.

The analysis demonstrated comparable improvements in alignment parameters between technology‐assisted and conventional techniques. Technology‐assisted procedures resulted in fewer HKA, LDFA and MPTA alignment outliers than did conventional methods. Component positioning showed favourable results for both femoral (LDFA) and tibial (MPTA) components, with a higher percentage achieving neutral alignment within ±3°. However, these differences in alignment did not consistently translate into superior clinical outcomes, as most studies reported comparable functional results between technology‐assisted and conventional techniques. Advancements in navigation and robotic systems—from first‐generation platforms to more sophisticated ones (e.g., MAKO, CORI)—demonstrate a trend towards improved alignment, precise positioning control, and enhanced evaluation of ligament balancing. The absence of consistent evidence supporting meaningful clinical benefits over conventional approaches underscores the need for cautious integration of these technologies into clinical practice. Patient‐specific factors and surgeon expertise remain critical in determining their appropriate application.

At the time of this report, the European Medicines Agency had yet to approve any RA surgical system for revTKA. However, the CORI Surgical System (Smith & Nephew) has received U.S. Food and Drug Administration (FDA) 510(k) clearance for revTKA procedures using its RA surgical platform. Notably, all technology platforms in the included studies were used off‐label for revision procedures because they were originally designed for primary arthroplasty. Recent FDA clearance for specific systems in revision settings is leading to the development of purpose‐built platforms optimised for the unique challenges of revision surgery. These emerging technologies may potentially translate into clinical benefits as designs evolve, surgical workflows are streamlined and longer‐term outcome data become available.

For primary TKA, the literature has shown that the improved accuracy achieved in primary RA TKA is not accompanied by significantly improved clinical outcomes compared with manual TKA [12]. Nevertheless, the added complexity and variability inherent to revision surgery may magnify the advantages of robotic technology, with potentially improved accuracy in revision implant positioning and ligament balancing which, in turn, could translate into better long‐term outcomes. Theoretically, unlike manual revTKA, bony cuts and alignment can be achieved independently of the anatomical variations present in the general population. Additionally, ligament status can be assessed more objectively, allowing for precise implant positioning and balancing, or aiding in decisions regarding implant constraint. Furthermore, data on positioning and ligament balancing can be correlated with functional outcomes and implant performance and survival, potentially identifying specific targets for optimal implant positioning and ideal ligament tension.

In revUKA, studies revealed no significant differences in clinical or radiographic outcomes when compared with conventional techniques or primary TKA. While similar technology‐assisted systems—particularly MAKO and Orthopilot—were commonly utilised in the included studies, the lack of homogeneity regarding indications, follow‐up durations, and comparison groups limited the feasibility of a direct quantitative synthesis. Moreover, studies with longer follow‐up periods did not consistently report a substantial reduction in complication rates, suggesting that the impact of technology assistance on reducing postoperative risks in revUKA may be limited. These results are consistent with previous meta‐analyses evaluating the impact of RA surgery in primary TKAs [40, 46], which reported fewer alignment outliers and more neutral postoperative HKA rates compared to conventional methods (odds ratio, 0.34; 95% CI, 0.20–0.58; *p* < 0.001), despite longer operative times [40]. Notably, the longer operative times did not lead to increased complication rates, suggesting that the additional surgical time may be an acceptable trade‐off for improved radiographic outcomes [40, 46].

The complication profiles between technology‐assisted and conventional approaches appeared comparable, although inconsistencies in reporting limit the ability to draw definitive conclusions. For revTKA, documented complications included wound healing issues, pin track complications associated with navigation, and mechanical issues requiring manipulation under anaesthesia. While specific complications such as pin track issues are unique to navigated cases, the current evidence suggests no major safety concerns with either approach [38].

While technology‐assisted revision knee arthroplasty offers precision benefits, its impact on surgical times remains unclear because of inconsistent reporting across studies and the absence of direct comparisons in the original articles. The included studies consistently indicated longer operative times when using computer navigation or robotic assistance in both revTKA and revUKA. For revTKA, additional operative times of 15–24 min were reported for navigated cases. In revUKA conversions, an interesting temporal trend was observed: earlier studies, such as that by Confalonieri et al. [10], reported substantial time differences of up to 44 min, whereas more recent studies utilising modern robotic platforms showed markedly reduced time differences (Tuecking et al., 6.4 min; [56] Yun et al, 2 min [62]). This trend may reflect a learning curve effect in computer‐assisted surgery. However, the significant variation in baseline operative times across studies likely reflects differences in case complexity, surgical team experience, and the specific platforms employed.

A recent systematic review and meta‐analysis concluded that conversions from UKA to TKA are associated with longer tourniquet times, poorer Western Ontario and McMaster Universities Arthritis Index pain and function scores, lower Oxford Knee Scores, and higher rates of reoperation compared with primary TKA [25]. In the current review, Mancino et al. [31], Lee et al. [24] and Tuecking et al. [56] reported comparable outcomes between revUKA and primary TKA, with no significant differences in clinical or radiographic outcomes. The disparity between the findings of specific studies in this review and those of the broader systematic analysis may be attributed to heterogeneity in surgical techniques, patient characteristics, or the type of technology assistance utilised in revUKA cases. Additionally, potential selection bias in smaller studies or variations in follow‐up durations could contribute to inconsistencies in reported outcomes.

The main limitations of this systematic review stem from the significant heterogeneity across the included studies regarding surgical indications, follow‐up durations, and the technological platforms employed, which ranged from basic navigation systems to advanced robotics. Methodological quality varied considerably, particularly among case series, with inconsistent reporting of complications and outcomes. This heterogeneity creates difficulty in drawing definitive conclusions on the added value of technology‐assisted versus conventional approaches. The absence of randomised controlled trials and the relatively small sample sizes in individual studies further weakened the strength of the evidence. Additionally, the evolution of technology over the study period introduced temporal biases that complicated direct comparisons. The lack of standardised reporting methods for surgical times, complications, and clinical outcomes further hindered a comprehensive analysis, particularly in evaluating the relationship between improved alignment and functional outcomes.

Looking forward, further high‐quality research is needed to clarify the potential advantages of technology‐assisted revision knee arthroplasty. Given the complexity and heterogeneity of the patient population undergoing revision knee arthroplasty, randomised controlled trials may not be applicable. Instead, larger cohort studies with consistent methodology and standardised reporting on surgical techniques, patient characteristics, and follow‐up durations could significantly contribute to the literature. Such studies would help delineate the relationship between improved alignment accuracy and long‐term outcomes, including prosthesis survival and patient‐reported satisfaction. Additionally, advancements in robotic and navigation platforms—along with customised (including three‐dimensionally printed) implants in cases with severe bone loss—may reduce operative times and improve accessibility, potentially enhancing their feasibility and cost‐effectiveness. The integration of machine learning and artificial intelligence could also play a pivotal role in optimising preoperative planning and intraoperative assessment of ligament status and bony defects, as well as decision‐making in implant positioning, selection, and fixation techniques, paving the way for more personalised surgical approaches. Finally, future studies must rigorously assess the cost‐effectiveness of these platforms, weighing initial acquisition costs and operating expenses against potential benefits. Institutions adopting these technologies should establish protocols for systematic outcome tracking to contribute to the growing evidence base.

## CONCLUSIONS

This systematic review demonstrates that technology‐assisted revision knee arthroplasty achieves improved alignment parameters and fewer outliers compared to conventional techniques, though these radiographic improvements do not consistently translate into superior clinical outcomes or reduced complication rates. While the evolution of navigation and robotic systems shows promising trends in alignment accuracy and surgical precision, the current evidence suggests that technology assistance should be carefully considered on a case‐by‐case basis, with attention to patient‐specific factors and surgeon expertise—particularly given the longer operative times and the lack of clear clinical benefits over conventional approaches.

## AUTHOR CONTRIBUTIONS

Gijs G. van Hellemondt contributed to the study conception and design. Screening and data collection were performed by Filippo Leggieri and Matteo Innocenti. Risk of bias assessment was conducted by Roberto Civinini and Filippo Leggieri. Data analyses were performed by Filippo Leggieri. Simon N. van Laarhoven and Tommy de Windt assessed the interpretation of the data. Gijs G. van Hellemondt supervised the study from inception. The first draft of the manuscript was written by Matteo Innocenti. Simon N. van Laarhoven and Tommy de Windt reviewed the draft, and all authors commented on previous versions of the manuscript. All authors read and approved the final manuscript.

## CONFLICT OF INTEREST STATEMENT

Gijs van Hellemondt reports a relationship with Smith and Nephew Inc that includes: consulting or advisory and speaking and lecture fees. Gijs van Hellemondt reports a relationship with Zimmer Biomet that includes: consulting or advisory and speaking and lecture fees. Simon van Laarhoven, Matteo Innocenti, and Roberto Civinini report consulting fees from Smith and Nephew Inc.

## ETHICS STATEMENT

The ethics statement is not available.

## Supporting information

Supporting information.

## Data Availability

The data sets used and/or analysed during the current study are available from the corresponding author on reasonable request.
