# Supplementary material for: Technology‐assisted revision knee arthroplasty reduces radiographic outliers compared with standard revision knee surgery: A systematic review
Source: Knee Surg Sports Traumatol Arthrosc. 2025 Jul 24;33(10):3606–20. doi: 10.1002/ksa.12748 (PMC12459324; doi:10.1002/ksa.12748)
Supplement: Supplementary file 1 — Supporting information. [file KSA-33-3606-s001.docx]

Support Materials

**Technology-Assisted Revision Knee Arthroplasty Reduces Radiographic Outliers Compared with Standard Revision Knee Surgery: A Systematic Review**

| PICOS question | |
| --- | --- |
| Population | Failed total knee replacement  Failed unicompartmental knee replacement |
| Intervention | Navigated or robotic-assisted revision knee arthroplasty |
| Comparison | Standard revision knee replacement |
| Outcomes | Clinical and radiological outcomes Complication rates |
| Study design | Randomized controlled trials (RCTs)  Cohort studies Case-control studies  Case series > 10 cases |

| MINORS Risk of bias assessment | | | | | | | | | | | | | |
| --- | --- | --- | --- | --- | --- | --- | --- | --- | --- | --- | --- | --- | --- |
|  | D1 | D2 | D3 | D4 | D5 | D6 | D7 | D8 | D9 | D10 | D11 | D12 | Overall |
| RevTKA | | | | | | | | | | | | | |
| Case series | | | | | | | | | | | | | |
| Cochrane, 2023 | 2 | 1 | 1 | 2 | 1 | 2 | 1 | 0 |  |  |  |  | 10 |
| Ochs, 2014 | 2 | 2 | 1 | 2 | 1 | 2 | 1 | 1 |  |  |  |  | 12 |
| Sikorski, 2004 | 0 | 1 | 1 | 1 | 1 | 1 | 1 | 1 |  |  |  |  | 7 |
| [Thielemann, 2007](https://pubmed.ncbi.nlm.nih.gov/?sort=date&size=200&term=Thielemann+FW&cauthor_id=17983115) | 1 | 1 | 1 | 1 | 1 | 1 | 1 | 1 |  |  |  |  | 8 |
| Cohorts studies | | | | | | | | | | | | | |
| MacAskill, 2024 | 2 | 1 | 2 | 1 | 1 | 2 | 1 | 0 | 0 | 0 | 0 | 2 | 12 |
| Massin, 2008 | 2 | 1 | 1 | 1 | 1 | 2 | 1 | 0 | 2 | 2 | 1 | 1 | 15 |
| Jenny, 2010 | 2 | 1 | 1 | 2 | 1 | 2 | 1 | 0 | 2 | 1 | 1 | 2 | 16 |
| Perlick, 2005 | 2 | 2 | 2 | 2 | 1 | 1 | 1 | 0 | 2 | 2 | 1 | 2 | 18 |
| [Hui-Ling Joanne Ngim, 2023](https://arthroplasty.biomedcentral.com/articles/10.1186/s42836-022-00160-5#auth-Hui_Ling_Joanne-Ngim-Aff1) | 2 | 1 | 2 | 2 | 1 | 2 | 1 | 0 | 2 | 1 | 2 | 2 | 18 |
| Cochrane, 2023 | 2 | 1 | 1 | 2 | 1 | 2 | 1 | 0 |  |  |  |  | 10 |
| RevUKA | | | | | | | | | | | | | |
| Case series | | | | | | | | | | | | | |
| Magruder, 2024 | 2 | 2 | 0 | 2 | 1 | 2 | 1 | 0 |  |  |  |  | 10 |
| Chatain, 2012 | 2 | 1 | 1 | 2 | 1 | 2 | 1 | 0 |  |  |  |  | 10 |
| Andriollo, 2024 | 2 | 1 | 2 | 2 | 2 | 2 | 2 | 0 |  |  |  |  | 13 |
| Cohorts studies | | | | | | | | | | | | | |
| Confalonieri, 2010 | 2 | 2 | 0 | 2 | 1 | 2 | 2 | 0 | 2 | 1 | 2 | 2 | 18 |
| Lachance, 2023 | 2 | 1 | 0 | 1 | 1 | 2 | 1 | 0 | 1 | 2 | 1 | 1 | 13 |
| Lee, 2018 | 2 | 1 | 0 | 2 | 1 | 2 | 1 | 0 | 0 | 1 | 2 | 2 | 14 |
| Tuecking, 2021 | 1 | 2 | 0 | 2 | 1 | 1 | 1 | 0 | 0 | 2 | 2 | 2 | 14 |
| Yun, 2020 | 1 | 1 | 0 | 1 | 1 | 2 | 1 | 0 | 2 | 1 | 1 | 2 | 13 |
| Saragaglia, 2015 | 2 | 1 | 0 | 2 | 1 | 2 | 1 | 0 | 2 | 0 | 1 | 2 | 14 |
| Mancino, 2024 | 2 | 1 | 2 | 2 | 1 | 2 | 1 | 0 | 0 | 2 | 2 | 2 | 17 |
